# Involvement of SYCP2L and TDRD3 gene variants on ovarian reserve and reproductive outcomes: a cross-sectional study

**DOI:** 10.5935/1518-0557.20220074

**Published:** 2023

**Authors:** Iasmim Ribeiro Rosa, Caio Parente Barbosa, Caroline Awoki Ferrandez, Bianca Del Bel Sonoda, Denise Maria Christofolini, Camila Martins Trevisan, Antonio Simone Laganà, Carla Peluso, Bianca Bianco

**Affiliations:** 1 Discipline of Sexual and Reproductive Health and Populational Genetics, Department of Collective Health, Faculdade de Medicina do ABC/Centro Universitário Saúde ABC, FMABC, Santo André, São Paulo, Brazil; 2 Instituto Ideia Fértil, Santo André, Brazil; 3 Unit of Gynecologic Oncology, ARNAS “Civico - Di Cristina - Benfratelli”, Department of Health Promotion, Mother and Child Care, Internal Medicine and Medical Specialties (PROMISE), University of Palermo, Palermo, Italy

**Keywords:** infertility, *in vitro* fertilization, single nucleotide variant, pharmacogenetic, ovarian reserve

## Abstract

**Objective:**

Single nucleotide variants have been implicated in the response to fertility treatment and a pharmacogenomic approach may help to customize therapy based on patient genome. We aimed to evaluate the effect, individual and combined, of SYCP2L (rs2153157:G>A) and TDRD3 (rs4886238:G>A) variants on ovarian reserve, response to controlled ovarian stimulation (COS) and reproductive outcomes of women undergoing in vitro fertilization (IVF) treatment.

**Methods:**

This cross-sectional study included 149 normoovulatory women undergoing IVF. Genotyping was performed using the TaqMan real-time polymerase chain reaction method. Clinical parameters and reproductive outcomes were compared according to the genotypes of the variants studied.

**Results:**

Considering ovarian reserve, there were no significant differences among *SYCP2L* or *TDRD3* genotypes in terms of FSH levels or AFC; however, AMH levels were significantly different in carriers of both variants. Regarding the *SYCP2L* rs2153157:G>A variant, lower AMH levels were observed in women carrying an AA genotype compared to women carrying a heterozygous genotype (*p*=0.01). Considering the *TDRD3* rs4886238:G>A variant, women carrying an AA genotype presented higher AMH levels than carriers of GG and GA genotypes (*p*=0.025). Nevertheless, no difference was found regarding response to COS or reproductive outcomes. Considering the combined effect of the variants, women carrying the heterozygous genotype of both variants presented statistically increased AMH levels compared to *SYCP2L* rs2153157 AA genotype carriers and *TDRD3* rs4886238 GG genotype carriers (*p*=0.042).

**Conclusions:**

Individually and combined, the *SYCP2L* rs2153157 and TDRD3 rs4886238 variants have an effect on AMH level.

## INTRODUCTION

It has been estimated that over 1.5 million in-vitro fertilization (IVF)/intracytoplasmic sperm injection (ICSI) cycles are performed annually worldwide, and this number increases each year (Dyer *et al*., 2016). The outcome of assisted reproduction is largely influenced by the effectiveness of controlled ovarian stimulation (COS) as well as ovarian reserve; however, the response to COS varies widely from woman to woman (Oehninger, 2011; Roque *et al.*, 2019).

**Table 1:** Genotype and allele frequencies of the SYCP2L and TDRD3 gene variants in the studied women.

Gene	Variant	Genotype			MAF	HWE
		WW	WV	VV		
*SYCP2L*	rs2153157:G>A	0 (0%)	49 (32.9%)	100 (67.1%)	0.165	0.090
*TDRD3*	rs4886238: G>A	79 (53%)	59 (39.6%)	11 (7.4%)	0.272	0.544

WW: wild-type genotype; WV: Heterozygous genotype; VV: variant genotype. MAF: Minor allele frequency; HWE: Hardy-Weinberg equilibrium.

**Table 2:** Clinical characteristics, hormonal profile and reproductive outcomes of normoovulatory infertile women.

Variables^*^	
N	149
Age (years old)	33.0 [32.0 - 34.0]
BMI	23.9 [23.3 - 24.5]
Menarche (years old)	13.0 [12.0 - 13.0]
Menstrual cycle interval (days)	28.0 [28.0 - 29.0]
Infertility duration (years)	3.0 [3.0 - 4.0]
Infertility cause (n, %) Male factor Tubo-peritoneal factor Idiopathic	90 (60.4%) 27 (18.1%) 32 (21.5%)
FSH (IU/mL)	6.1 [5.8 - 6.5]
LH (IU/mL)	2.9 [2.7 - 3.2]
Progesterone (ng/mL)	7.7 [4.2 - 10.2]
Prolactin (ng/mL)	16.0 [14.0 - 17.0]
AMH (ng/mL)	3.3 [2.9 - 3.8]
AFC (min-max)	9.0 [8.0 - 10.0]
COS protocol 100/150 UI 200 UI	90 (60.4%) 59 (39.6%)
Response to COH Poor Satisfactory Hyper-response	32 (21.5%) 97 (61.5%) 20 (13.4%)
Oocytes retrieved	6.0 [5.0 - 7.0]
MII Oocytes	5.0 [4.0 - 6.0]
Embryos	3.0 [2.0 - 4.0]
Pregnancy rate	46 (30.9%)

* These variables were shown as median and CI (95%): 95% Confidence Interval; BMI: body mass index; COH: controlled ovarian hyperstimulation; FSH: follicle stimulating hormone; LH: luteinizing hormone; AMH: anti-Müllerian hormone; AFC: antral follicle counting; MII: metaphasis II oocytes.

**Table 3:** Clinical characteristics, hormonal profile and reproductive outcomes of normoovulatory infertile women according to rs2153157:G>A variant of the SYCP2L gene.

Variables	Genotypes *SYCP2L* rs2153157:G>A			*p*-value
	GG	GA	AA	
N	0	49	100	-
Age (years)	-	32 (31-33)	33 (32-34)	0.39^a^
BMI (kg/m^2^)	-	23.9 (22.5-24.9)	24.0 (23.3-24.8)	0.54^a^
Menarche (years old)	-	13.0 (12.0-13.0)	13.0 (12.0-13.0)	0.98^a^
Menstrual cycle interval (days)	-	28 (28-30)	28 (28-29)	0.40^a^
Infertility duration (years)	-	3.0 (2.1-4.0)	3.0 (3.0-4.0)	0.15^a^
FSH (UI/mL)	-	6.1 (5.7-6.7)	6.1 (5.7-6.6)	0.72^a^
LH (UI/mL)	-	3.2 (2.6-3.5)	2.8 (2.7-3.2)	0.30^a^
Progesterone (ng/mL)	-	4.1 (1.2-11.0)	8.2 (5.6-11.0)	0.53^a^
Prolactin (ng/mL)	-	15.0 (14.0-19.4)	16.0 (13.0-17.7)	0.33^a^
AMH (ng/mL)	-	3.7 (3.2-5.8)	2.9 (2.4-3.6)	0.01^a^
AFC	-	9 (8-10)	9 (8-11)	0.47^a^
COS protocol (n, %) 100 UI 150 UI 200 UI	- - -	26 (53.1%) 4 (8.2%) 19 (39.8%)	47 (47.0%) 13 (13.0%) 40 (40.0%)	0.63^b^
Response to COS (n, %) Poor Satisfactory Hyper-response	- - -	13 (26.5%) 27 (55.1%) 9 (18.4%)	19 (19.0%) 70 (70.0%) 11 (11.0%)	0.19^b^
Oocytes retrieved	-	6 (4-7)	6 (5-7)	0.48^a^
MII Oocytes	-	5.0 (3.0-6.9)	5.0 (4.0-6.0)	0.39^a^
Embryos	-	3 (2-4)	3 (2-4)	0.55^a^
Pregnancy rate (n, %)	-	17 (34.7%)	29 (29.0%)	0.48^b^

The variables were shown as median and CI (95%): 95% Confidence Interval; BMI: body mass index; COH: controlled ovarian hyperstimulation; FSH: follicle stimulating hormone; LH: luteinizing hormone; AMH: anti-Müllerian hormone; AFC: antral follicle counting; MII: oocytes metaphasis II.

a Mann-Whitney Test and

b Chi-squared test.

**Table 4:** Clinical characteristics, hormonal profile and reproductive outcomes of normoovulatory infertile women according to rs4886238:G>A variant of the TDRD3 gene.

Variables	Genotypes TDRD3 rs4886238:G>A			*p*-value
	GG	GA	AA	
N	79	59	11	-
Age (years)	33.0 (32.0-34.0)	32.0 (31.0-33.5)	33.0 (29.7-36.0)	0.943^a^
BMI (kg/m^2^)	23.4 (23.0-24.4)	24.2 (22.8-25.0)	25.3 (22.9-27.5)	0.363^a^
Menarche (years old)	13.0 (12.0-13.0)	13.0 (12.0-13.0)	13.0 (11.4-14.0)	0.873^a^
Menstrual cycle interval (days)	28.0 (28.0-29.0)	28.0 (28.0-29.0)	29.0 (27.4-30.0)	0.859^a^
Infertility duration (years)	3.0 (3.0-4.0)	3.0 (3.0-4.0)	3.0 (2.0-6.3)	0.946^a^
FSH (UI/mL)	6.1 (5.7-6.8)	6.0 (5.7-6.4)	6.2 (4.2-7.3)	0.888^a^
LH (UI/mL)	3.0 (2.7-3.4)	2.9 (2.6-3.3)	2.8 (2.1-3.4)	0.826^a^
Progesterone (ng/mL)	8.1 (3.7-11.0)	5.8 (2.0-10.8)	7.3 (1.0-13.2)	0.868^a^
Prolactin (ng/mL)	14.8 (12.4-16.9)	16.9 (14.0-19.4)	18.9 (9.0-24.9)	0.377^a^
AMH (ng/mL)	2.8 (2.0-3.6)	3.4 (3.0-4.9)	4.5 (1.7-7.9)	0.025^a^
AFC	9 (8.0-10.0)	9.0 (8.0-10.0)	9.0 (7.7 -12.4)	0.858^a^
COS protocol (n, %) 100 UI 150 UI 200 UI	39 (49.4%) 7 (8.9%) 33 (41.8%)	28 (47.5%) 9 (15.2%) 22 (37.3%)	6 (54.5%) 1 (9.1%) 4 (36.3%)	0.814^b^
Response to COS (n, %) Poor Satisfactory Hyper-response	16 (20.2%) 51 (64.5%) 12 (15.2%)	11 (18.6%) 41 (69.5%) 7 (11.8%)	5 (45.4%) 5 (45.4%) 1 (9.1%)	0.343^b^
Oocytes retrieved	5.0 (5.0-7.0)	6.0 (5.0-9.0)	4.0 (0.0-6.3)	0.139^a^
MII Oocytes	6.0 (4.0-6.7)	5.0 (4.0-7.5)	4.0 (0.0-6.0)	0.201^a^
Embryos	2.0 (2.0-4.0)	3.0 (2.0-4.0)	2.0 (0.0-3.3)	0.182^a^
Pregnancy rate (n, %)	24 (30.4%)	21 (35.6%)	1 (9.1%)	0.215^b^

The variables were shown as median and CI (95%): 95% Confidence Interval; BMI: body mass index; COH: controlled ovarian hyperstimulation; FSH: follicle stimulating hormone; LH: luteinizing hormone; AMH: anti-Müllerian hormone; AFC: antral follicle counting; MII: oocytes metaphasis II.

a Mann-Whitney Test and

b Chi-squared test.

The ovarian reserve declines differently in each woman. Besides, the ovarian reserve tests used to date have only modest-to-poor predictive properties (Broekmans *et al.*, 2006; Broer *et al*., 2013; Amanvermez & Tosun, 2016; Peluso *et al*., 2021). COS is the first step in every IVF/ICSI cycle and aims to allow the development and maturation of multiple follicles and oocytes, thus increasing cumulative pregnancy rates from IVF. However, some patients present an unexpected hyporesponse or even a hyper-response to gonadotropin stimulation, which can cause adverse events leading to physical and psychological distress (Roque *et al*., 2019). Consequently, research has been conducted to find reliable predictors of effective COS and good ART outcomes.

Individual genetic variability is known to affect ovarian reserve and the outcome of COS, and a pharmacogenomic approach may help to customize therapy based on patient genome (Roque *et al.*, 2019; Trevisan *et al.*, 2019). Variants in different genes, such as single nucleotide polymorphisms (SNP) or single nucleotide variants (SNV), have been implicated in the response to fertility treatment.

The most studied SNPs are *FSHR:*c.919G>A*, FSHR:*c.2039G>A and *FSHR:*c.-29G>A, which were previously associated with variability in serum FSH level and reproductive outcomes (Pabalan *et al.*, 2014; Alviggi *et al.*, 2016; Santi *et al*., 2018). Nonetheless, many other gene variants have been studied and appear to influence the IVF outcomes. Laisk-Podar *et al.* (2015) searched for genetic markers of ovarian function, ovarian stimulation and IVF treatment outcomes among genetic variants related to female reproductive ageing in Estonian patients. The results revealed that among 36 variants searched, the rs2153157 of the *SYCP2L* gene was associated with amount of recombinant FSH (rFSH) and chance of biochemical and clinical pregnancy. In addition, rs4886238 of the *TDRD3* gene was associated with both the number of punctured ovarian follicles and oocytes retrieved.

In this scenario, the aim of this study was to analyze the individual and combined effects of *SYCP2L* (rs2153157) and *TDRD3* (rs4886238) variants on ovarian reserve, response to COS, and reproductive outcomes of Brazilian women undergoing IVF treatment.

## MATERIAL AND METHODS

### Patients

A cross-sectional study was performed between September 2016 and September 2019 and included 149 normoovulatory Brazilian women undergoing IVF treatment at the Instituto Ideia Fertil - Human Reproduction and Genetics Center of the School of Medicine of the ABC, Santo Andre, Brazil. The Research Ethics Committee of the School of Medicine of the ABC approved the study design (certificate CAAE 64167716.9.1001.0082) and all participants signed informed consent terms before joining the study to allow anonymized data collection for purposes of research.

The investigation into the cause of infertility included hormonal and biochemical profiling, testing for sexually transmitted diseases, imaging examinations, investigation of genetic and/or immunological abnormalities, hysterosalpingography, hysteroscopy, laparoscopy, and partner semen analysis.

The inclusion criteria were as follows: age ≤38 years old; FSH ≤12.0 IU/L; TSH >0.5 to <4 IU/L; serum prolactin <25 ng/mL; body mass index (BMI) >18.5 to ≤30; ovulatory cycles during 25-35 days; presence of both ovaries without morphological abnormalities; and having no evidence of endocrine disease. Patients with endometriosis, polycystic ovarian syndrome, previous ovarian surgery or chemo/radiotherapy, and couples whose male partner underwent invasive procedures for sperm retrieval were excluded.

### Antral follicle count

Ovaries were evaluated before initiation of COS on the second day of the menstrual cycle by transvaginal ultrasonography using a conventional two-dimensional transvaginal ultrasound at 7MHz (Philips^®^). The antral follicle count (AFC) was performed on each ovary and was considered as follicles counted up to 2-10 mm (Broekmans *et al*., 2010).

### Sample collection

Peripheral blood samples of 15 mL were collected in a tube containing clot-separator gel and in a tube containing ethylenediaminetetraacetic acid (EDTA). After collection, the tubes for biochemical tests were centrifuged at 1000 rpm for 10 min; plasma was aliquoted into microtubes and frozen at -80°C for further determination of hormone concentrations. The tube for DNA extraction was stored at 6°-8°C until extraction.

### Hormone level measurements

Follicle-stimulating hormone (FSH), luteinizing hormone (LH), and anti-Müllerian hormone (AMH) levels were measured during the early follicular phase of the menstrual cycle. Progesterone and prolactin were measured during the luteal phase of the menstrual cycle. FSH, LH, progesterone, and prolactin were measured with an enzyme-linked fluorescent immunoassay (BioMerieux^®^, Hazelwood, MO) and AMH Gen II was measured with an enzyme-linked immunosorbent assay (Beckman Coulter^®^, Inc., Brea, CA).

### IVF treatment

COS was performed using exogenous recombinant FSH (rFSH, Puregon^®^) at a fixed dose per day administered on average for 8 to 14 days, starting on the second or third day of the menstrual cycle. GnRH antagonist (Orgalutran^®^) was administered when the follicles reached a diameter of approximately 14 mm, until the largest follicles reached between 17 and 20 mm, as measured in transvaginal ultrasonography. At this time, the patient was given chorionic gonadotropin (hCG-Choriomon^®^) at a dose of 5000 IU or recombinant HCG (Ovidrel^®^, Merck S/A, 250 mg/0,5 mL). After 34-36 h, transvaginal ultrasound-guided follicular puncture for oocyte retrieval was performed (Barbosa *et al.*, 2014).

The classification of the response to COS was as follows: i) satisfactory response characterized by the development of ≥4 to ≤15 follicles larger than 14 mm after 6 days of ovarian stimulation with gonadotropins; ii) hyper response and/or ovarian hyperstimulation syndrome (OHSS) characterized by multiple ovarian follicles (>15 follicles) together with potential clinical symptoms, such as ascites, hematological changes (hemoconcentration), pleural effusion, and liver and/or coagulation abnormalities; and iii) poor response characterized by the development of up to three follicles smaller than 14mm (Polyzos & Sunkara, 2015).

A maximum of two embryos were transferred on the third or fifth day after IVF/ICSI. Luteal phase support was carried out with vaginal progesterone at a dose of 600 mg/day starting on the day of oocyte retrieval. Pregnancy was confirmed by b-hCG measurement (>25 mIU/mL) on Day 12 after embryo transfer.

### Genotyping

Genomic DNA was extracted from lymphocytes using the salting out method (Lahiri & Numberger, 1991). Genotyping of the variants *SYCP2L*:g.10897255G>A (chr6:10897255, NC_000006.12:g.10897255G>A, rs2153157) and *TDRD3:*g.60539605G>A (chr13:60539605, NC_000013.11:g.60539605G>A, rs4886238) was performed using the TaqMan system by real-time polymerase chain reaction according to manufacturer instructions (ThermoFisher Scientific^®^, Waltham, MA).

### Statistical Analyses

Descriptive analyses were performed based on absolute and relative frequencies for categorical variables, and on medians with a 95% confidence interval for quantitative variables. Data distribution was analyzed with the Shapiro-Wilk test. Hardy-Weinberg equilibrium of the variants studied was verified using the Chi-squared test. The Mann-Whitney test was used to analyze the effect of each variant on clinical characteristics, hormone levels and reproductive outcomes. The chi-squared test was used to certify the associations between the variants and the response to COS, rFSH protocol, and pregnancy rate. The Kruskal-Wallis test was used followed by Dunn’s test to analyze the difference in the combined genotype of the rs4886238 and rs2153157 variants in hormonal profile and reproductive outcomes. Statistical analyses were performed with Stata^®^ software (SE 11.0) for Windows and significance was considered at *p*<0.05.

## RESULTS

Of the 149 women, the genotype distribution for *SYCP2L* rs2153157:G>A variant was 0% (0/149) carriers of wild-type homozygous genotype (GG), 32.9% (49/149) heterozygous (GA) and 67.1% (100/149) carriers of the variant homozygous genotype (AA). The G and A allele were frequent in 16.5% and 83.5% of the women, respectively. Considering the *TDRD3* rs4886238:G>A variant, the genotype distribution was 53% (79/149) wild-type homozygous genotype (GG), 39.6% (59/149) heterozygous and 7.4% (11/149) variant homozygous genotype (AA). The wild-type G allele was found in 72.8% of women, while the variant allele G was found in 27.3%. Both *SYCP2L* rs2153157:G>A and *TDRD3* rs4886238:G>A variants were in Hardy-Weinberg equilibrium, *p*=0.090 and *p*=0.544, respectively.

The clinical characteristics, hormonal profile and reproductive outcomes of the women studied are shown in Table 1. Of the 149 normoovulatory women undergoing IVF treatment, 65.1% (97/149) had satisfactory response to COS, 21.5% (32/149) had poor response, and 13.4% (20/149) had hyper-response. Eleven of the 20 hyper-responders presented minor symptoms of ovarian hyperstimulation syndrome (OHHS), such as ovarian enlargement, abdominal bloating, and pain (OHSS grade 1), without significant changes in renal or hepatic function until clinical improvement was achieved. Thirty-one patients had no embryo to transfer: 21 had their cycles canceled due to lack of ovarian response or monofollicular response (14.1% of the IVF cycles); nine had no embryo development; and one patient had empty follicle syndrome. Therefore, the pregnancy rate was calculated based on 118 patients undergoing embryo transfer; 39.0% (46/118) of these patients had positive b-HCG test results.

The clinical characteristics, hormonal profile and reproductive outcomes of the women were compared according to *SYCP2L* (Table 2) and *TDRD3* (Table 3) genotypes. Age, BMI, menarche, menstrual cycle interval, and infertility duration did not differ between the genotypes for either *SYCP2L* or *TDRD3* variants. Considering ovarian reserve, there were no significant differences between the *SYCP2L* or *TDRD3* genotypes in terms of FSH levels and AFC. However, AMH levels were significantly different between variants. In the *SYCP2L* rs2153157:G>A variant, lower AMH levels were observed in women carrying the homozygous variant genotype (AA) compared to women carrying the heterozygous genotype (3.7 ng/mL *versus* 2.9ng/mL, *p*=0.01). In the *TDRD3* rs4886238:G>A variant, women carrying the homozygous variant genotype (AA) presented higher AMH levels compared to GG and GA genotypes (4.5 ng/mL, 2.8 ng/mL, and 3.4 ng/mL, respectively, *p*=0.025). Nevertheless, no difference was found in reproductive outcomes.

Considering the combined effect of the genotypes of the *SYCP2L* rs2153157:G>A and *TDRD3* rs4886238:G>A variants, 41.6% (n=62) of the patients presented the *SYCP2L*:AA/*TDRD3*:GG genotypes; 20.1% (n=30) the *SYCP2L*:GA*/TDRD3:*AA genotypes; 19.4% (n=29) the *SYCP2L*:GA*/TDRD3:GA* genotypes, and 18.8% (n=28) other combinations of genotypes. Only AMH level was different according to combination of genotypes (*p*=0.031) (Table 4). Women carrying the heterozygous genotype (GA) of both variants presented statistically higher AMH levels compared to women carrying the homozygous variant genotype (AA) of the *SYCP2L* rs2153157 and wild-type homozygous genotype (GG) of the *TDRD3* rs4886238 variant [3.5 (3.0-6.9) ng/mL *versus* 2.8 (1.9-3.5) ng/mL, *p*=0.042].

## DISCUSSION

Despite the growing data about IVF around the world, no biomarker has been described as an accurate predictor of response to COS or IVF reproductive outcome. Use of SNPs that can predict the effectiveness of drugs in individual patients, depending on their genetic background, may add further elements in this direction (Ramaraju *et al.*, 2018).

During oogenesis, homologous chromosomes are paired for recombination between chromosomes, mediated by the formation of a synaptonemic complex, a tripartite structure made up of two lateral/axial elements and a central element (Costa & Cooke, 2007; Yang & Wang, 2009). In rats, the SYCP2 protein is a component of the lateral/axial element (Schalk *et al*., 1998). In mammals, SYCP2L is a homologous sequence of SYCP2. In Xenopus oocytes, the SYCP2L protein is exclusively expressed in immature oocytes (Voltmer-Irsch *et al.*, 2007), with *Sycp2* -/- knockout mice showing accelerated loss of oocytes and reduced fertility, evidencing their role in the regulation of the survival of primordial oocytes (Schramm *et al.*, 2011; Zhou *et al.*, 2015).

The diplotene stage in oocytes extends from birth to ovulation, lasting up to four decades in women. The SYCP2L protein in oocyte centromeres is involved in the organization of the local chromatin around the centromeres and may play an important role in sensing and repairing DNA damage to promote oocyte survival (Zhou *et al*., 2015). Recent genome-wide association studies (GWAS) showed that variants of the human *SYCP2L* locus have been associated with age at menopause (He *et al*., 2009; Carty *et al*., 2013).

The human *SYCP2L* gene is located at 6p24.2 and the variant g.10897488G>A (rs2153157) is located at intron 4. Zhou *et al.* (2015) found that variant allele A changes the splicing efficiency and may therefore regulate the steady-state amount of SYCP2L transcript. The A allele is spliced more efficiently than the G allele, and is thus expressed at a higher level. The authors demonstrated that SYCP2L promotes the survival of reserve oocytes and regulates reproductive aging in females.

The *TDRD3* gene (Tudor domain-containing protein 3) is located on chromosome 13q21.2 and is a transcriptional co-activator and regulator of estrogen-mediated gene transcription. In addition, it interacts with the FMRP protein (Fragile X protein) involved in the development of primary ovarian failure (Linder *et al.*, 2008; Sullivan *et al.*, 2011), which suggests a role in ovarian reserve.

In the study of Laisk-Podar *et al.* (2015), women carrying the AA genotype of the *SYCP2L* rs2153157:G>A variant needed less rFSH to obtain an oocyte and had greater chances of attaining biochemical and clinical pregnancy. The authors srtressed that these results may indicate that carriers of the AA genotype have greater ovarian reserve. In addition, the authors suggested that the positive effect of the variant allele in clinical pregnancy rates may result directly from having a larger ovarian reserve or be associated with the role that the synaptonemal complex plays in preventing chromosome segregation errors and embryo aneuploidy, one of the main causes of implantation failure and early miscarriage. Regarding the *TDRD3* rs4886238:G>A variant, COS resulted in more follicles and oocytes in women carrying the G allele. The authors stressed that previously the G allele had been associated with earlier menopause (Stolk *et al.*, 2012) and this finding along with their results indicate that the ovarian pool is depleted more quickly in women carrying the G allele.

In the present study, none of the included women had the GG genotype of the *SYCP2L* rs2153157:G>A variant. Although the occurrence the minor G allele was lesser than in different genetic databases (Reference SNP Report - rs2153157, 2021), the frequency of the genotypes was in Hardy-Weinberg equilibrium in the studied population. Unlike Laisk-Podar *et al.* (2015), in our study the A allele was not associated with having a larger ovarian reserve, since ovarian reserve marker such as age, FSH and AFC were not different between carriers of G or A alleles. The A allele variant was not significantly associated with AMH levels, whereas women with the AA genotype presented significantly lower AMH levels in comparison to women with the GA genotype. Nevertheless, pregnancy rates were not different between alleles. Regarding the *TDRD3* rs4886238:G>A variant, women carrying the AA genotype presented statistically higher AMH levels than their counterparts with the GA and GG genotypes (Table 3). However, no differences were observed in ovarian reserve tests or reproductive outcomes. The combined effect of the *SYCP2L* rs2153157:G>A and the *TDRD3* rs4886238:G>A variants was different only to AMH levels, whereas women with the heterozygous genotype (GA) of both variants presented statistically higher AMH levels compared to women with the homozygous variant genotype (AA) of the *SYCP2L* rs2153157 and wild-type homozygous genotype (GG) of the *TDRD3* rs4886238 variant.

The different findings between studies can be attributed to several factors: patient selection criteria; sample size; ovarian stimulation protocol; and differences in ethnicity, all of which hinder the interpretation of the results. The Brazilian population was built with contributions from Europeans, Africans and Amerindians, resulting in a highly heterogenous genetic profile, the likes of which seldom seen in other parts of the world (Gaspar Neto & Santos, 2011; Salzano & Sans, 2014; Ramos *et al.*, 2016). Due to genetic diversity, the Brazilian population may show different allele frequencies than those presented in non-mixed populations. Nevertheless, Brazil is not represented in genomic datasets, such as gnomAD and TOPMed, although these databases have recently included Latin American samples (Naslavsky *et al.*, 2022).

AMH is considered the most sensitive ovarian reserve test (Tal & Seifer, 2017). It is strongly correlated with the primordial follicle pool and has an inversely proportional correlation with age (Kelsey *et al.*, 2012). Revelli *et al.* (2016) observed that in women with very low AMH levels, the ones who achieved clinical pregnancy had AMH levels comparable to those who did not achieve pregnancy. Peluso *et al*. (2021) evaluated age, FSH, AMH, AFC, and the ovarian response prediction index (ORPI), as potential predictors of response to COS, and found that none of them individually or combined showed good predictive capacity for hypo-response. The authors also found that AMH alone was the best predictor for hyper-response, while the ORPI demonstrated the best predictive capacity. Indeed, it is still debatable whether AMH might be considered a reliable marker of IVF outcomes (Peñarrubia *et al*., 2005; Fiçicioglu *et al.*, 2006; Lekamge *et al.*, 2007; Broer *et al*., 2011; Siddiqui *et al*., 2019; Peluso *et al.*, 2021).

## CONCLUSION

In conclusion, *SYCP2L* rs2153157 and *TDRD3* rs4886238 variants individually have an effect on AMH levels, whereas the homozygous variant genotype AA was associated with lower AMH levels for the *SYCP2L* rs2153157:G>A variant and higher AMH levels for the *TDRD3* rs4886238:G>A variant. No differences were found in other markers of ovarian reserve, response to COS, or reproductive outcomes in the genotypes of the studied variants. The combined effects of the *SYCP2L* rs2153157 and *TDRD3* rs4886238 variants also have an effect on AMH levels, while women carrying the combination of GA genotypes of both variants presented higher AMH levels.
